# Effects of a sodium fluoride- and phytate-containing dentifrice on remineralisation of enamel erosive lesions—an in situ randomised clinical study

**DOI:** 10.1007/s00784-018-2351-z

**Published:** 2018-02-08

**Authors:** Jonathan E. Creeth, Charles R. Parkinson, Gary R. Burnett, Susmita Sanyal, Frank Lippert, Domenick T. Zero, Anderson T. Hara

**Affiliations:** 1 GSK Consumer Healthcare, St George’s Avenue, Weybridge, Surrey KT13 0DE UK; 2 Syneos Health, Hyderabad, India; 3 Oral Health Research Institute, Indiana University School of Dentistry, Indianapolis, IN USA

**Keywords:** Dental erosion, Demineralisation, Dentifrice, Fluoride, Polyphosphate, Remineralisation

## Abstract

**Objective:**

The objective of this work was to evaluate effects of a dentifrice containing sodium fluoride (1150 ppm F) and the organic polyphosphate phytate (0.85% *w*/*w* of the hexa-sodium salt) on in situ remineralisation of early enamel erosive lesions and resistance to subsequent demineralisation.

**Materials and methods:**

Subjects (*n* = 62) wore palatal appliances holding eight bovine enamel specimens with pre-formed erosive lesions. They brushed their natural teeth with the phytate test dentifrice (TD); a positive control dentifrice (PC, 1150 ppm fluoride as NaF); a reference dentifrice (RD, disodium pyrophosphate + 1100 ppm fluoride as NaF) or a negative control dentifrice (NC, fluoride-free) in a randomised, double-blind, crossover design. Specimens were removed at 2, 4 and 8 h post-brushing and exposed to an ex vivo acid challenge. Surface microhardness (Knoop) was measured at each stage. The primary efficacy variable was relative erosion resistance (RER); other variables included the surface microhardness recovery (SMHR), acid resistance ratio (ARR) and enamel fluoride uptake (EFU).

**Results:**

After 4 h, the results for RER, ARR and EFU were in the order PC > TD = RD > NC with PC > TD = RD = NC for SMHR. Results at 2 and 8 h were generally consistent with the 4 h data. Mineralisation progressed over time. Dentifrices were generally well-tolerated.

**Conclusions:**

In this in situ model, addition of phytate or pyrophosphate to a fluoride dentifrice inhibited the remineralising effect of fluoride. Both formulations still delivered fluoride to the enamel and inhibited demineralisation, albeit to a lesser extent than a polyphosphate-free dentifrice.

**Clinical relevance:**

Addition of phytate or pyrophosphate to a fluoride dentifrice may reduce its net anti-erosive properties.

## Introduction

Dental erosion is the loss of tooth substance due to chemical dissolution by acids of non-bacterial origin [1], which causes enamel to soften and become more susceptible to abrasive wear [2, 3]. In the initial stages, the eroded enamel surface can be repaired through replacement of lost mineral structure by salivary calcium and phosphate [4, 5]. In a series of in situ clinical studies, dentifrice formulations containing up to 1426 ppm fluoride as sodium fluoride (NaF) have been shown to accelerate remineralisation and enhance acid resistance of the enamel surface [6–8]. A significant dose–response for fluoride has been observed [7, 8]. Interestingly, certain dentifrices were significantly less effective than others in terms of promoting enamel remineralisation and erosion resistance, even though fluoride concentrations were matched. This suggests that non-fluoride ingredients in the dentifrice formulation may modulate fluoride effects on the remineralisation and demineralisation process [7].

Cosmetic benefits of dentifrice formulations are also important, particularly their ability to remove extrinsic tooth stains. Whilst abrasives are almost universally added to dentifrices to achieve this, a balance must be reached between adequate stain removal during toothbrushing and potential of abrasives to increase wear of the dental hard tissues over the lifetime of the tooth [9, 10]. An approach to improve stain control without substantially increasing abrasivity is to include polyphosphate salts in the formulation [10, 11]. Linear condensed polyphosphates bind to tooth surfaces and complex calcium ions, interfering with the development and retention of extrinsic stains [11, 12], as well as dental calculus [13]. Whilst published long-term clinical studies suggest that polyphosphates do not impair the anticaries benefits of fluoride [14, 15], linear polyphosphates can inhibit the exchange of mineral ions on the tooth surface, thereby interfering with the ability of fluoride to remineralise eroded enamel [7, 16, 17].

Phytate (myo-inositol hexakis [dihydrogen phosphate]) is an organic cyclic polyphosphate ion without direct phosphate–phosphate bonds. It is a potential alternative to established inorganic linear condensed polyphosphates for dentifrice applications. In vitro, phytate has been shown to rapidly absorb onto hydroxyapatite and is a potent dissolution inhibitor [18]. Phytate also modifies the permselectivity of enamel and dentine, with potential benefits in terms of caries progression [19]. In early animal caries studies, phytate was shown to inhibit demineralisation and exhibit anticaries effects in the absence of fluoride [20]. Furthermore, a clinical study of a phytate-containing mouthwash demonstrated that phytate is able to reduce dental calculus formation [21], but there is otherwise little recent published information on oral applications of this molecule. The substantially different structure of phytate versus linear condensed polyphosphates may mean it interacts with enamel surfaces in a different way, potentially able to provide stain removal benefits without the degree of influence on remineralisation processes.

The aim of this work was to investigate the influence of phytate in a dentifrice on intra-oral enamel remineralisation and subsequent resistance to demineralisation. An in situ clinical study was performed to examine the effects of an experimental fluoride dentifrice formulation containing phytate (0.85% *w*/*w*) on (bovine) enamel remineralisation, enamel fluoride uptake and resistance to acid challenge after a single brushing. Performance was determined relative to a positive control (fluoride but no phytate), a negative control (no phytate or fluoride) and a reference dentifrice (containing fluoride and an alternative polyphosphate). To make inferences regarding potential differences in the time-course of these effects, results were obtained after 2, 4 and 8 h of intraoral exposure [22, 23].

## Materials and methods

This was a single-centre, randomised, examiner- and analyst-blind, crossover study performed at the Oral Health Research Institute (OHRI) of the Indiana University School of Dentistry, Indianapolis, IN, USA. The protocol was approved by the Indiana University Institutional Review Board (IRB #1506930286), and the study was conducted in accordance with the Declaration of Helsinki, the International Conference on Harmonisation of Technical Requirements for Registration of Pharmaceuticals for Human Use and local laws and regulations. All subjects provided written informed consent prior to screening, demonstrated understanding of the protocol and were considered willing, able and likely to comply with all study procedures. There was one minor administrative amendment to the protocol that did not affect study flow or outcomes.

### Study population

Subjects aged 18–65 years previously accepted into the OHRI dental erosion in situ panel and for whom a palatal appliance had been constructed were screened for this study. Subjects were eligible for inclusion if they had an unstimulated salivary flow rate of ≥ 0.2 mL/min and a stimulated salivary flow rate of ≥ 0.8 mL/min; a maxillary dental arch suitable for retention of the palatal appliance and good general and oral health. Subjects were excluded from participation if they were pregnant or breastfeeding; had a medical history that could prevent them from participating in the study until the study conclusion; had any sign of grossly carious active lesions, moderate or severe periodontal conditions, or severe tooth wear; wore an oral appliance or orthodontia; were receiving any medication that could interfere significantly with salivary flow; had an intolerance or hypersensitivity to the study materials; or had used any investigational products or participated in another clinical trial within 30 days of the screening visit.

### Study treatments

Details of study dentifrices are listed in Table 1.

**Table 1:** Study treatments

Dentifrice	Fluoride^a^ (ppm)	Phytate^b^ (% *w*/*w*)	KNO_3_ (% *w*/*w*)	Stain removal agents
Test	1150	0.85	5.0	Hydrated silica, hexasodium phytate
Positive control^c^	1150	–	5.0	Hydrated silica
Reference^d^	1100	–	–	Hydrated silica, disodium pyrophosphate
Negative control	–	–	5.0	Hydrated silica

KNO_3_, potassium nitrate; ppm, parts per million; *w*/*w*, weight-for-weight

^a^As sodium fluoride (NaF)

^b^% w/w of hexasodium salt

^c^Sensodyne® Pronamel®—Mint Essence; GSK Consumer Healthcare, Weybridge, UK (US-marketed dentifrice)

^d^Crest 3D White Luxe™ Glamorous White™; Procter & Gamble, Cincinnati, OH, USA (US-marketed dentifrice)

### Experimental design

At the screening visit, subjects underwent examination of the oral hard tissue and oral soft tissue (OST) and were assessed for eligibility. Eligible subjects had their palatal appliance fitted for comfort and were provided with a fluoride-free dentifrice to use for 2 days before each treatment visit. OST examinations were also performed at the beginning of each treatment visit.

Subjects were instructed to follow their usual oral hygiene habits over the course of the study, with the exception that they were required to use the fluoride-free washout toothpaste for 2 days before each visit and were not permitted to brush their teeth or use any fluoride-containing products on the morning of any visit. Subjects could eat breakfast on the morning of each visit, as long as this was at least 30 min prior to the visit.

At the baseline visit, eligible subjects were randomised according to a schedule generated in advance by the Biostatistics Department of GSK Consumer Healthcare. Each subject completed all treatments in a random order, one at each treatment visit. The study dentifrices were supplied in over-wrapped tubes. The site personnel, study statistician and other employees of the study site or sponsor who had the potential to influence study outcomes were blinded to product allocation.

At each of the four treatment visits, a study dentist placed the subject’s palatal appliance holding the eight bovine tooth enamel specimens in the subject’s mouth. After an equilibration period of at least 5 min, the subject was provided with a toothbrush (Sensodyne® Soft; GSK Consumer Healthcare, Weybridge, UK) loaded with 1.5 g of the assigned dentifrice. A study technician instructed the subjects to brush the buccal surfaces of their natural teeth for 25 timed seconds to create a slurry, then swish the slurry around the palatal appliance for 1 min and 35 s (timed) to permit direct contact with the enamel specimens. Subjects expectorated the slurry, then gently rinsed with 15 mL of deionised water for 10 s and expectorated the rinse.

After completing the brushing/rinsing procedures, subjects continued to wear their palatal appliance for a total of 8 h, with a single interruption of 30 min after 4 h, during which they could eat a meal and drink bottled water (Ice Mountain® Spring Water, Nestlé Waters North America Inc., Stamford, CT, USA) (> 0.1 ppm F). Subjects were instructed to refrain from talking for the first hour after brushing and were not permitted to drink water for the first 2 h of the test period, but they could drink water after this under the supervision of study personnel. Subjects were not permitted to leave the study site or sleep when the appliance was in their mouth.

Enamel specimens were removed in a predetermined order from the appliance at 2 h (two specimens, one each from the end of the left- and right-side specimen holder, respectively), 4 h (four specimens, two from the centre of each specimen holder) and 8 h (remaining two specimens).

### In situ erosion remineralisation model

This study used an in situ erosion–remineralisation model developed by Zero et al. [6]. In brief, bovine enamel blocks were polished flat and the enamel side of the specimen was ground until the enamel surface had a minimum 3 × 3 mm facet in the centre. The enamel specimens were immersed in commercially available grapefruit juice for 25 min then rinsed thoroughly with deionised water. Following sterilisation with ethylene oxide, the demineralised enamel blocks were fixed to a tailored intra-oral palatal appliance engineered to hold eight specimens mounted on two plastic holders. The palatal appliance was inserted into the subject’s mouth for 5 min prior to treatment to allow a degree of pellicle formation. The appliance remained in the subject’s mouth for 8 h following treatment, with the enamel specimens removed at predefined intervals as described above. After the specimens were removed from the appliance, they underwent a second demineralisation treatment using the procedure described above.

Changes in the mineral content of the enamel specimens were evaluated using the surface microhardness (SMH) test [6, 24, 25]. Five indentations were made in the centre of each enamel specimen using a Knoop diamond (2100 HT; Wilson Instruments). Indentation lengths were measured, and the mean indentation length was calculated. Indentation lengths were determined prior to the first in vitro erosive challenge (B), after the first in vitro erosive challenge (E1), after the treatment-induced in situ remineralisation phase (R) and after the second in vitro erosive challenge (E2).

### Data analysis

The extent of remineralisation was calculated as the percent SMH recovery (%SMHR), based on the following formula: %SMHR = [(E1-R) / (E1-B)] ∗ 100 [26].

Overall enamel resistance to erosive challenge was calculated as the percent relative erosion resistance (%RER) based on the following formula: %RER = [(E1-E2) / (E1-B)] ∗ 100 [8, 27].

The acid resistance of the enamel specimens following intra-oral exposure to the study dentifrices was calculated as the acid resistance ratio (ARR) based on the following formula: ARR = 1-[(E2-R) / (E1-B)] (based on Creeth et al. [8]).

Enamel fluoride uptake (EFU) during the remineralisation phase was determined using the microdrill enamel biopsy technique following the in situ remineralisation phase and SMHR test and before the second in vitro erosive challenge [28]. Enamel specimens were drilled to a depth of approximately 100 μm through the entire lesion (four cores per specimen). EFU was expressed as μg F per cm^2^ of enamel surface.

These analyses were performed on the enamel specimens after 2, 4 and 8 h of intra-oral remineralisation. An exploratory analysis was performed to determine whether any change with time in the SMHR, RER, ARR or EFU endpoints was a function of treatment: this was called the “time-by-treatment” analysis.

### Safety

Adverse events (AEs) and any abnormalities in the OST examination were recorded from the start of the study period until 5 days after the last administration of study product. Clinical judgement was exercised by the investigator to diagnose the AE and to assess the relationship between the study product and the occurrence of each AE, with intensity graded as mild, moderate or severe.

### Statistical analysis

This study aimed to recruit up to 62 subjects to ensure that at least 56 subjects completed all study visits. This sample was calculated to have 80% power to detect a difference in %RER of approximately 10.7 between products, assuming two-sided paired tests at a 5% significance level. A difference of 10.7 in %RER was considered as modest to be detected between not only the Test dentifrice and fluoride-free Negative control but also between Positive control and Reference formulations containing fluoride. In a study of similar design, the standard deviation (SD) for the difference between a test dentifrice and a reference dentifrice was 28.4 for %RER [8].

Analysis of covariance (ANCOVA) was used to analyse the primary efficacy variable (%RER) and the other secondary response variables. The ANCOVA model included a random effect for subject and fixed effects for study period and treatment with subject- and period-level baseline, and subject and period level pre-treatment covariates. Pair-wise comparisons between the treatments on all endpoints were performed. All tests were two-sided at a 5% significance level.

The exploratory endpoint (differences in the time-course of remineralisation between treatments on %RER, %SMHR, ARR and EFU) was analysed using an ANCOVA model with %RER, %SMHR, ARR and EFU as dependent variables, fixed effect as treatment, period, time of extraction and a time-by-treatment interaction as fixed effect and subject as random effect. Baseline and pre-treatment acid challenge values were included as covariates.

Efficacy analyses were conducted on the per-protocol (PP) population, defined as all subjects who were randomised into the study, received at least one dose of study product, had at least one post-baseline efficacy assessment and had no protocol violations deemed to affect efficacy during the study. The intent-to-treat (ITT) population was defined as all subjects who were randomised into the study, received at least one dose of study product and had at least one post-baseline efficacy assessment. Efficacy analyses were also to be performed on the ITT population if there was more than a 10% difference in the number of subjects in the ITT and PP populations. The safety population included all randomised subjects that were dispensed the study treatment at least once during the study.

## Results

### Subjects

**Fig. 1:**
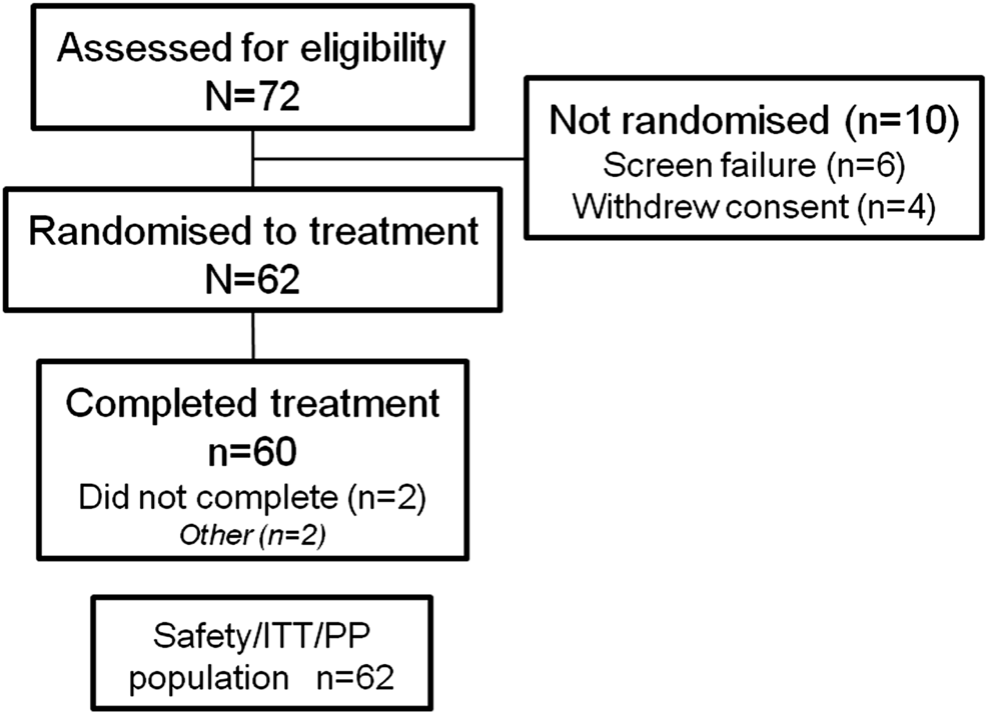
Subject flow diagram. ITT, intent-to-treat; PP, per-protocol

The first subject was enrolled on 24 August 2015, and the last subject completed the study on 18 November 2015. Of the 72 subjects screened, 62 were randomised to treatment and 60 completed the study (Fig. 1). All randomised subjects were included in the ITT and safety populations. Subjects were aged 19 to 63 years (mean 41.0; SD 11.35), and 67.7% were female. The majority of subjects were white (62.9%), with 30.6% identifying as black/African American and 6.5% as Asian.

### Efficacy

The raw mean SMH values for the enamel specimens at each stage of the different treatments are summarised in Table 2.

**Table 2:** Enamel microhardness mean indent lengths (μm, ± standard error) as a function of treatment, stage of the experiment, and duration of remineralisation

Time point	Treatment	No. of subjects	B		E1		R		E2	
			Mean indent length	S.E.	Mean indent length	S.E.	Mean indent length	S.E.	Mean indent length	S.E.
2 h	Test	62	43.42	0.100	60.06	0.217	57.66	0.232	69.90	0.341
	Positive control	62	43.30	0.108	59.93	0.185	55.87	0.212	64.92	0.411
	Reference	61	43.42	0.113	59.88	0.232	57.97	0.209	70.21	0.339
	Negative control	62	43.26	0.120	60.27	0.196	57.72	0.220	73.84	0.408
4 h	Test	62	43.38	0.101	60.12	0.181	57.13	0.195	69.38	0.288
	Positive control	62	43.24	0.107	59.98	0.182	55.45	0.187	64.86	0.269
	Reference	60	43.37	0.102	59.86	0.180	57.38	0.204	69.42	0.297
	Negative control	62	43.23	0.106	59.87	0.181	56.94	0.209	72.25	0.398
8 h	Test	62	43.41	0.098	60.06	0.224	56.41	0.247	67.80	0.342
	Positive control	62	43.32	0.117	59.99	0.242	54.27	0.238	63.81	0.333
	Reference	60	43.30	0.130	60.00	0.257	56.74	0.235	68.70	0.361
	Negative control	62	43.17	0.124	59.70	0.224	56.08	0.249	70.14	0.408

### RER

**Fig. 2:**
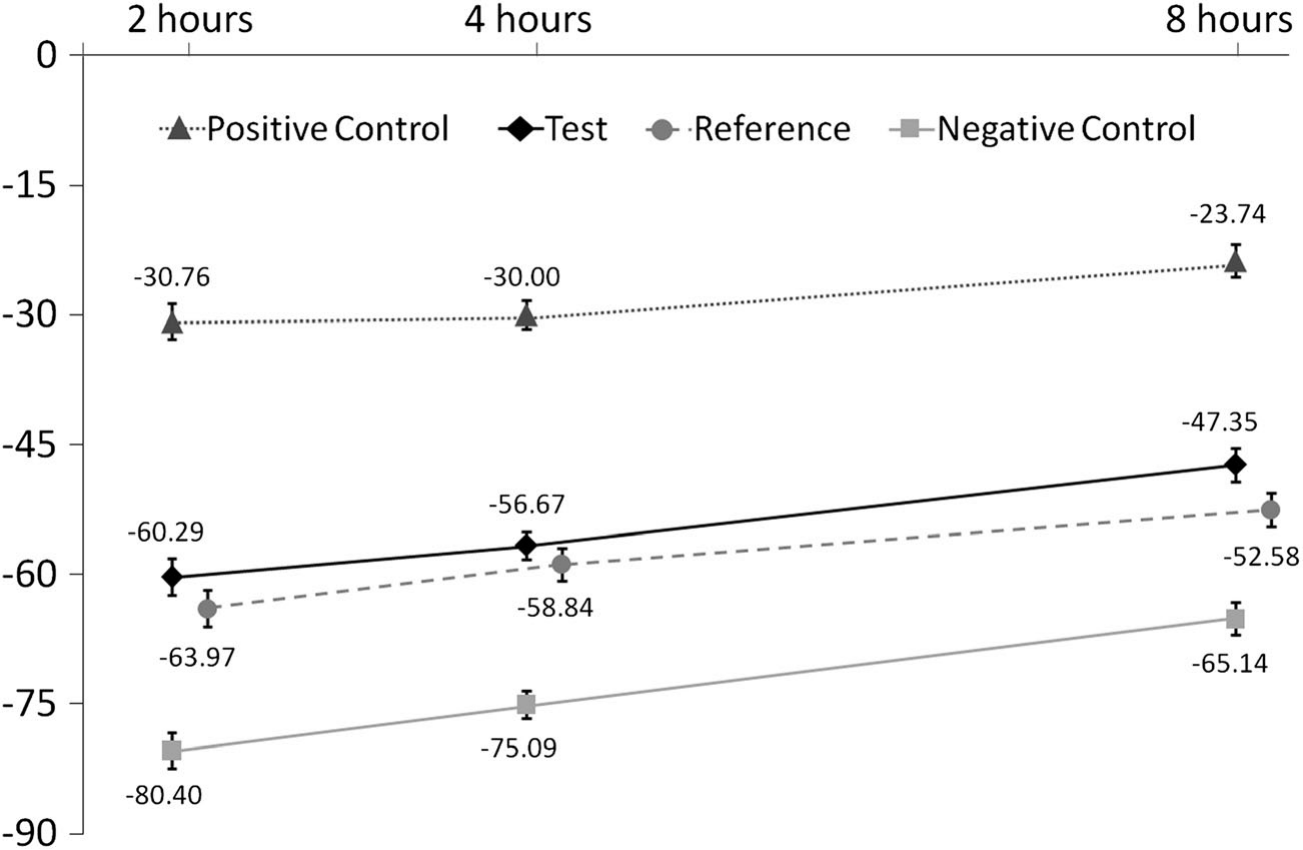
Percent relative acid resistance (%RER) versus specimen removal time (adjusted means ± standard error). Data for the Reference dentifrice are offset for clarity

Adjusted mean %RER (± standard error [SE]) at 2, 4 and 8 h for each dentifrice is shown in Fig. 2. At the 4-h time point (see Table 3, showing treatment differences, 95% confidence intervals [CI] and *p* value), the RER value for the experimental phytate formulation (Test dentifrice) was higher than that of the Negative control by 18.4 percentage points (*p* < 0.0001), but there was no significant difference between the Test and Reference dentifrices. The RER values for the Positive control were 45.1 and 26.7 percentage points higher than those of the Negative control and Test dentifrice, respectively (both *p* < 0.0001). The RER for the Reference dentifrice was significantly higher than that of the Negative control dentifrice (*p* < 0.0001).

**Table 3:** Treatment comparisons at 4 h post-treatment (mean difference with higher and lower confidence intervals)

Treatment comparison	%RER	%SMHR	ARR	EFU
	Difference^a^ (95% CI) *p* value			
Test vs. Pos cont	−26.67 (−31.10, −22.30) **< 0.0001**	−9.24 (−11.70, −6.74) **< 0.0001**	−0.17 (−0.21, −0.13) **< 0.0001**	−0.66 (−0.88, −0.45) **< 0.0001**
Test vs. Neg cont	18.42 (14.03, 22.82) **< 0.0001**	−0.61 (−3.12, 1.89) 0.6287	0.19 (0.15, 0.23) **< 0.0001**	0.93 (0.71, 1.15) **< 0.0001**
Test vs. Ref	2.17 (−2.24, 6.58) 0.3330	2.50 (−0.01, 5.01) 0.0509	0.00 (−0.04, 0.04) 0.8811	0.21 (−0.01, 0.43) 0.0574
Pos cont vs. Neg cont	45.09 (40.69, 49.49) **< 0.0001**	8.63 (6.12, 11.13) **< 0.0001**	0.36 (0.32, 0.40) **< 0.0001**	1.59 (1.38, 1.81) **< 0.0001**
Pos cont vs. Ref	28.84 (24.40, 33.28) **< 0.0001**	11.74 (9.21, 14.27) **< 0.0001**	0.17 (0.13, 0.21) **< 0.0001**	0.88 (0.66, 1.09) **< 0.0001**
Ref vs. Neg cont	16.25 (11.81, 20.69) **< 0.0001**	−3.12 (−5.64, −0.59) **0.0160**	0.19 (0.15, 0.23) **< 0.0001**	0.72 (0.50, 0.94) **< 0.0001**

RER, relative erosion resistance; SMHR, surface microhardness recovery; ARR, acid resistance ratio; EFU, enamel fluoride uptake; Pos cont, Positive control; Neg cont, Negative control; Ref, Reference

Statistically significant comparisons are highlighted in bold

^a^Difference is first-named treatment minus second-named treatment; positive difference favours first-named treatment

Similar differences and statistical significances were also demonstrated at 2 and 8 h post intra-oral exposure except that the difference in RER for the Test dentifrice versus the Reference dentifrice became statistically significant (*p* = 0.0444) after 8 h exposure.

There was a consistent upward trend for the %RER values as a function of time for all treatments. There was no indication that the rate of rise was a function of treatment: the time-by-treatment interaction effect size was 1.04 (*p* = 0.3968).

### SMHR

**Fig. 3:**
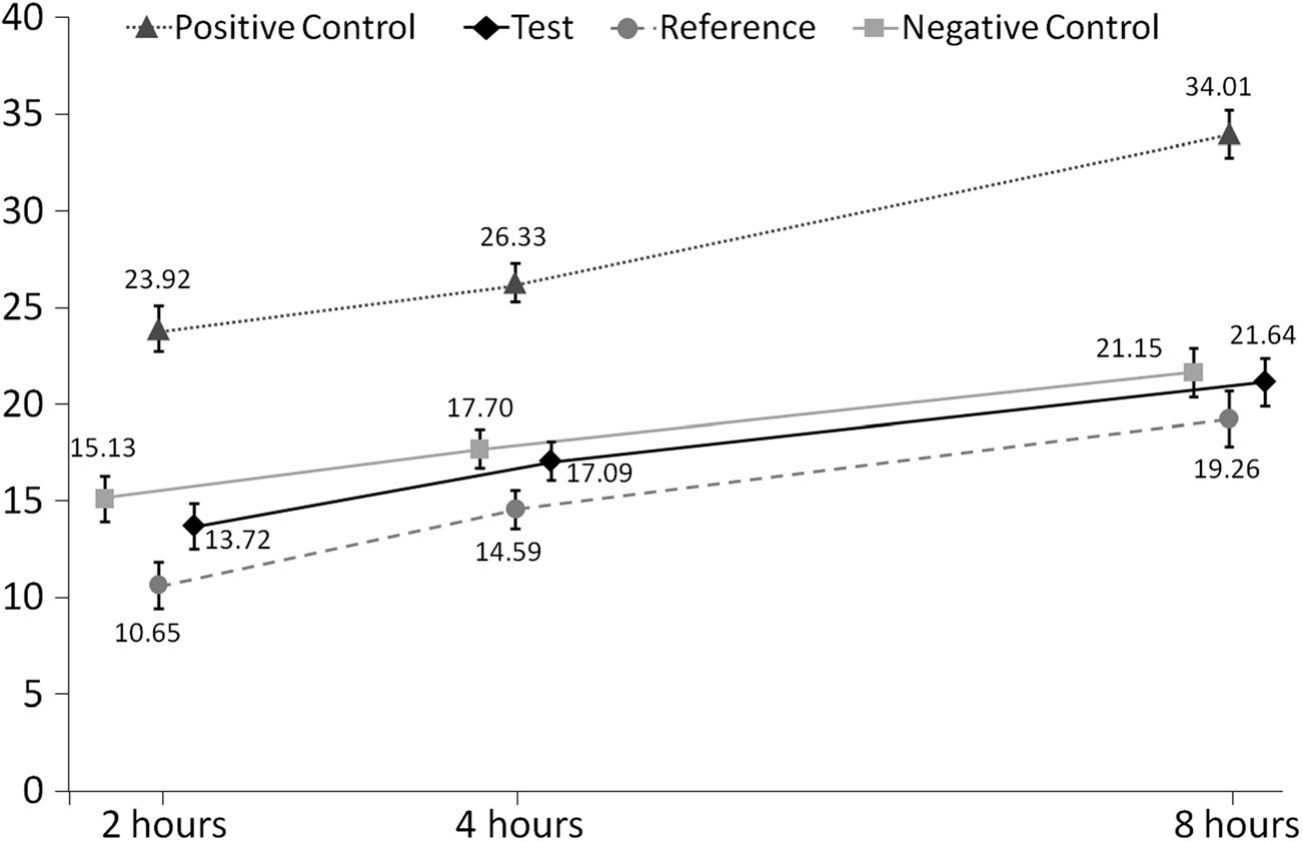
Percent surface microhardness recovery (%SMHR) versus specimen removal time (adjusted means ± standard error). Data for the Negative Control and Test dentifrices are offset for clarity

Adjusted mean %SMHR (± SE) at 2, 4 and 8 h for each dentifrice is shown in Fig. 3. At the 4-h time point (see Table 2), there was no detectable increase in SMHR for the Test dentifrice versus the Negative control or Reference dentifrice. In contrast, the SMHR value for the Positive control dentifrice was 8.63 percentage points higher than that of the Negative control and was statistically significantly superior to all other dentifrices (all *p* < 0.0001). The SMHR value for the Reference dentifrice was slightly but significantly lower than that for the Negative control (difference of −3.1%; *p* = 0.0160).

Similar differences and statistical significances were demonstrated at 2 and 8 h post intra-oral exposure, except that no statistically significant differences between the Reference dentifrice and the Negative control were seen at 8 h.

As for RER, there was a consistent upward trend for the SMHR values as a function of time for all treatments. Again, there was no indication that the rate of rise was a function of treatment: the time-by-treatment interaction effect size was 0.84 (*p* = 0.5413).

### ARR

**Fig. 4:**
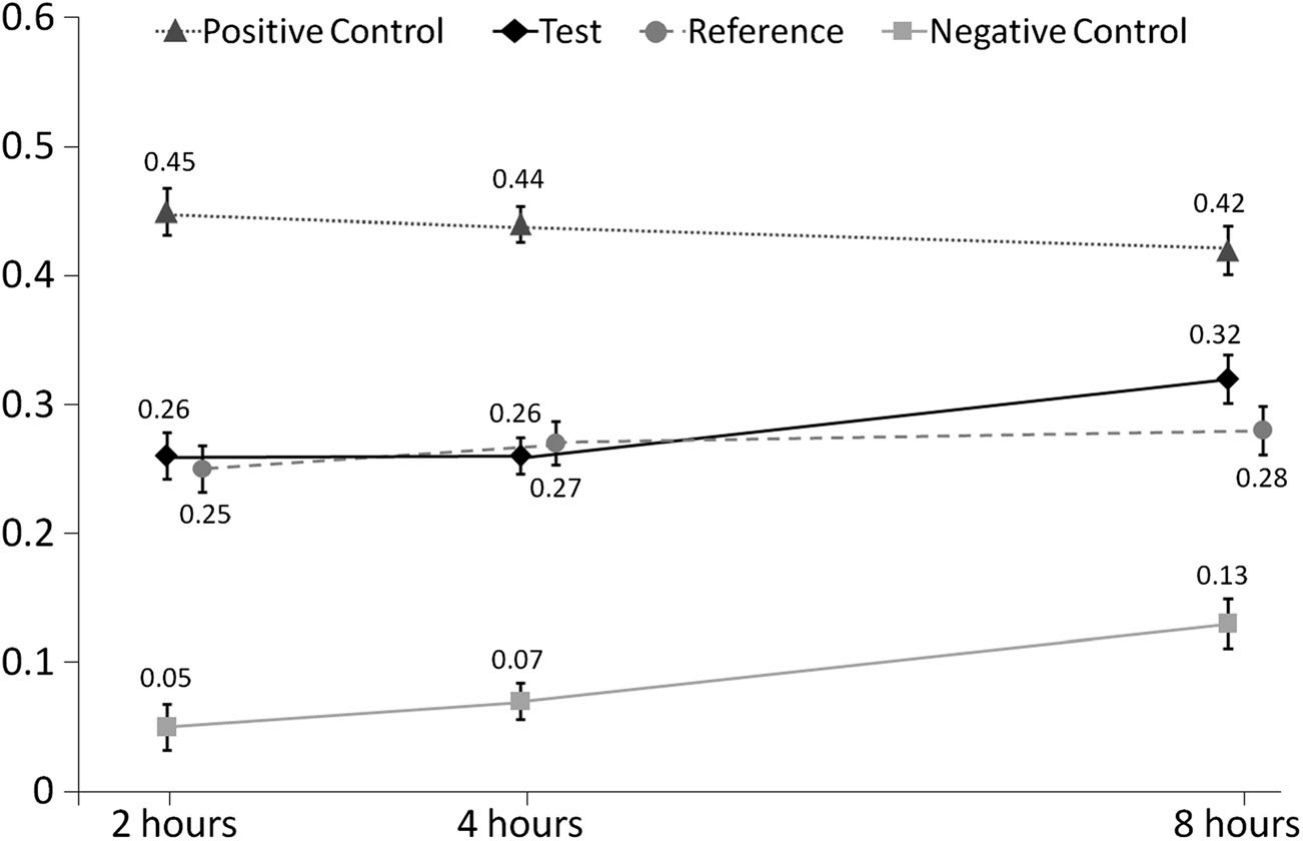
ARR, acid resistance ratio versus specimen removal time (adjusted means ± standard error). Data for the Reference dentifrice are offset for clarity

The ARR values at 2, 4 and 8 h for each dentifrice treatment are shown in Fig. 4. At the 4-h time point (see Table 3), there was a difference of 0.19 in ARR between the Test dentifrice and the Negative control (*p* < 0.0001) but there was no significant difference between the Test and Reference dentifrice. The Positive control dentifrice gave statistically significantly greater ARR compared to all other dentifrices (*p* < 0.0001 for all), including a difference of 0.17 compared to the Test dentifrice. Similar differences and statistical significances were also demonstrated at 2 and 8 h post intra-oral exposure.

The change in ARR value with time was found to be a function of applied treatment: the time-by-treatment interaction was statistically significant (at the 10% level generally accepted for such interaction effects) with an effect size of 2.11 (*p* = 0.0506). This appeared to be driven particularly by the ARR value for the Negative control, which was highest at 8 h, and the ARR value for the Positive control, which was lowest at this time point.

### EFU

**Fig. 5:**
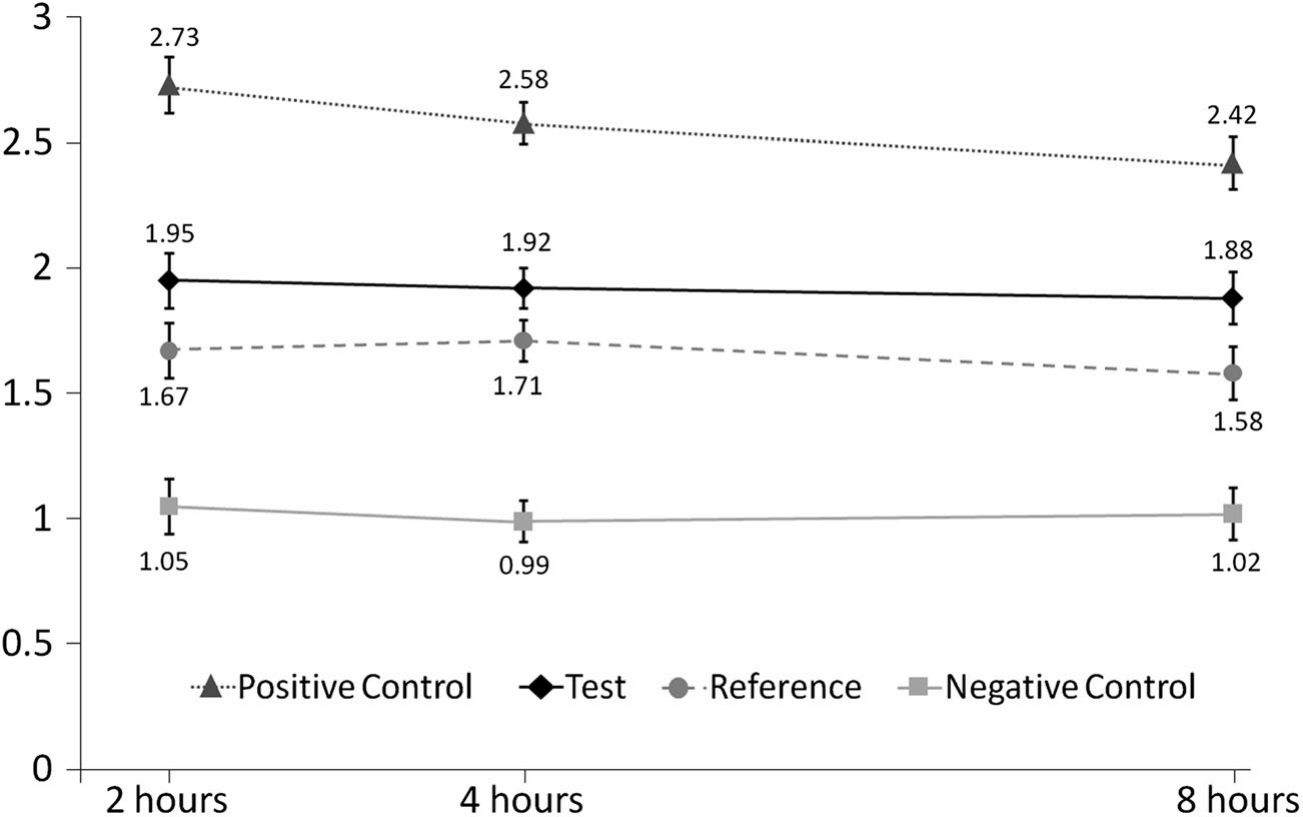
Enamel fluoride uptake versus specimen removal time (adjusted means ± standard error).

Adjusted mean EFU at 2, 4 and 8 h for each dentifrice is shown in Fig. 5. At the 4-h time point (see Table 2), the Test dentifrice gave statistically significantly higher EFU than the Negative control (*p* < 0.0001), but there was no significant difference between the Test and Reference dentifrices. The Positive control gave significantly higher EFU than all other dentifrices (*p* < 0.0001 for all), and there was a significant difference favouring the Reference dentifrice compared to the Negative control (*p* < 0.0001).

Similar differences and statistical significances were demonstrated at 2 and 8 h post intra-oral exposure for most of the comparisons except between the Test and Reference dentifrices at 8 h, favouring the Test dentifrice (difference of 0.30 [95% CI 0.01, 0.59]; *p* = 0.0425).

In contrast to the RER and SMHR profiles, there was a consistent downward trend for the EFU values as a function of time, most clearly for the Positive control treatment. However, there was no indication of a time-by-treatment interaction for EFU (effect size of 0.54; *p* = 0.7766).

### Safety

Treatment-emergent AEs were reported in eight subjects receiving the Test dentifrice (10 events, six of which were oral), six subjects receiving the Positive control (six events, three oral), two subjects receiving the Reference dentifrice (two events, both oral) and six subjects receiving the Negative control (seven events, three oral). No AEs were considered treatment-related, and no serious AEs occurred.

## Discussion

This randomised, controlled clinical study used an in situ enamel erosion model to explore the effects of phytate on fluoride-mediated enamel rehardening of acid-softened enamel and subsequent protection against an acid challenge. This model has been used previously to demonstrate that single brushing with fluoride dentifrices can remineralise eroded enamel and protect it against subsequent acid challenge [6–8, 22, 25].

The use of bovine enamel as a surrogate for human enamel, as in this study, has been reviewed by Lippert et al. [29]. Bovine enamel is advantageous in that it provides a large, flat surface with uniform enamel thickness and it comes from a source that has not been previously exposed to cariostatic levels of fluoride, or a diet as varied as is common for human teeth. Disadvantages of using bovine enamel include that there are differences in porosity, carbonate and fluoride content surface hardness, which mean that demineralisation progresses more quickly than in human enamel [29]. However, a recent study comparing fluoride dose–response of caries lesions in bovine and human enamel found that although demineralisation was higher in the former, the response to fluoride was similar [30].

A limitation of this model is that it does not factor in the impact of the dentifrice abrasive during application (as normally occurs with toothbrushing), or the potential impact of other abrasive surfaces, such as the tongue. This is intentionally done to isolate the remineralising effects of the dentifrices in the short-term period: this study was concerned with biochemical effects of the dentifrice on enamel surfaces, rather than physical effects. It is possible that mechanical contact could wear away part of the demineralized enamel layer, not allowing enough time for full fluoride remineralisation. Further studies with modified in situ methodologies would be required to understand the potential impact of physical wear on remineralisation. Laboratory studies in which abrasivity and fluoride concentration have been varied systematically have shown the ability of free fluoride ion to protect against abrasive wear from toothbrushing [31].

In the present study, the in situ model demonstrated the expected effect of the Positive control fluoride dentifrice on both promoting remineralisation (as measured by SMHR) and protecting against subsequent demineralisation (as measured by ARR) versus the fluoride-free Negative control. The RER measure, which incorporates both remineralisation and demineralisation in the calculation, was similarly supportive of fluoride performance in this model.

However, this study showed that, using this model, incorporating phytate in a dentifrice (at 0.85% *w*/*w* hexasodium salt) completely inhibited the remineralising effect of fluoride. When phytate was present, fluoride uptake was also reduced, but by no means eliminated, and the formulation still provided significant protection against acid challenge. The retention of this demineralisation-protective effect was sufficient to keep the RER value for the experimental phytate product still greater than that of the fluoride-free control. The level of fluoride uptake appears sufficient to explain the size of the demineralisation-protective effect observed; there is little suggestion in the data that the phytate is providing significant acid protection. This result in an enamel erosion model does not reflect the existing data from the enamel caries field, where—in fluoride-free systems—phytate has been shown to be able to inhibit enamel demineralisation and have potential anticaries activity [18–20]. Further investigation is required to determine if phytate’s action at the enamel surface contributes to protection from dietary acid attack.

Similar results were obtained with the Reference pyrophosphate-containing formulation as seen with the Test phytate formulation. Whilst there was a range of differences between these two formulations that could influence remineralisation–demineralisation effects to a degree, it is reasonable to conclude that polyphosphates as a class, whether linear or cyclic, can exert a substantially negative impact on the remineralisation effect of fluoride ion in early enamel erosive lesions. This effect has been observed for pyrophosphate dentifrices in early enamel lesions in related in situ models [7, 16, 17]. It may be that any polyphosphate that can achieve multi-point contact with a hydroxyapatite crystal structure can inhibit mineral deposition into that structure from saliva.

The effect of intra-oral remineralisation time on the different parameters was modest but nevertheless intriguing: whilst remineralisation showed a clear upward trend through the time-course of the treatment, as measured by SMHR, the amount of fluoride in the enamel appeared to slowly drop (for the more effective treatments at least). That is, in this experiment, fluoride levels in the enamel surface were strongly boosted by the fluoride treatments but slowly decreased over time. Such a trend has been seen previously in this model, when enamel samples were removed from the in situ model between 5 min and 4 h after brushing with a fluoride dentifrice [23].

The observation that SMH increases whilst enamel fluoride content decreases may illustrate the dynamic nature of the enamel surface: ions at the surface can diffuse into and out of the surface, according to the balance of concentration of the ions in saliva and the affinity for those ions within the crystal structure. The presence of high levels of fluoride in the enamel surface after treatment will enhance the affinity of that surface for calcium and phosphate ions in saliva [32], tending to drive these ions into the surface, thereby increasing its SMH. Note also that lesions exposed to the oral cavity under the conditions of this model will naturally tend to remineralise on exposure to saliva (as shown by the results for fluoride-free control).

Most of the fluoride delivered into the oral cavity after brushing with fluoride toothpaste remains unbound. It is rapidly lost due to rinsing of oral surfaces, either with water at the end of brushing, or by saliva in the minutes afterwards: only a small fraction becomes bound to oral surfaces [33]. Of the proportion directly taken up by enamel, the majority will initially be in the form of non-specifically adsorbed fluoride [34]. Some of this will be lost again (washed away by saliva), and some will slowly convert to fluoridated apatite [34]. CaF_2_ is not expected to form, as [F] is too low and pH too high [35].

Furthermore, fluoride bound to soft tissues and plaque after brushing will slowly be released into the oral fluids. This fluoride will also promote enamel fluoride uptake and remineralisation, before it too is washed away by the flow of saliva. The observed outcome on SMH recovery and EFU is the sum of this complex set of competing processes. Further study of the time dependence of SMHR versus EFU is warranted.

This in situ study provides intriguing insights into the ability of fluoride dentifrices of different base formulations to influence enamel remineralisation–demineralisation processes after erosive attack, within the limitations of this in situ methodology. The lesions studied were very early, reactive erosive lesions: more established lesions may behave differently. Only a single concentration of phytate was studied; other concentrations may exert contrasting effects on intra-oral remineralisation and/or demineralisation. Furthermore, the single-use protocol leaves unanswered the question of what might happen to pre-eroded enamel surfaces if the test products were used over a longer time-period, with many cycles of erosive attack interleaved with periods of remineralisation.

In summary, this single-use in situ study in early enamel erosive lesions demonstrated that a dentifrice containing the cyclic, organic polyphosphate ion phytate (at 0.85% *w*/*w* hexasodium salt), in addition to 1150 ppm fluoride as NaF, can exert positive effects on the remineralisation–demineralisation processes at the surface of early enamel erosive lesions as measured by RER. However, no benefits for including phytate were detected: fluoride-induced remineralisation was strongly inhibited, and there was no evidence of an enamel protective benefit from phytate. Similar results were obtained for a formulation containing the conventional linear inorganic polyphosphate pyrophosphate and a matched level of NaF.

The clinical implications of these findings are that formulation ingredients can interfere with the enamel protective effects of fluoride. However, longer-term studies of the effects of polyphosphates in dentifrices, incorporating multiple remineralisation–demineralisation cycles, would be useful in determining the longer-term consequences of the intra-oral enamel surface chemistry observed in this study.
